# Changes in HIV Outcomes Following Depression Care in a Resource-Limited Setting: Results from a Pilot Study in Bamenda, Cameroon

**DOI:** 10.1371/journal.pone.0140001

**Published:** 2015-10-15

**Authors:** Bradley N. Gaynes, Brian W. Pence, Julius Atashili, Julie K. O’Donnell, Alfred K. Njamnshi, Mbu Eyongetah Tabenyang, Charles Kefie Arrey, Rachel Whetten, Kathryn Whetten, Peter Ndumbe

**Affiliations:** 1 Department of Psychiatry, University of North Carolina School of Medicine, Chapel Hill, NC, United States of America; 2 Department of Epidemiology, University of North Carolina Gillings School of Global Public Health, Chapel Hill, NC, United States of America; 3 University of Buea, Buea, Cameroon; 4 Department of Internal Medicine (Neurology Unit), the University of Yaoundé I, Yaoundé, Cameroon; 5 HIV/AIDS Treatment Center, Bamenda Hospital, Bamenda, Cameroon; 6 Regional Technical Group for the fight against HIV North West region, Bamenda Hospital, Bamenda, Cameroon; 7 Duke Global Health Institute, Duke University, Durham, NC, United States of America; 8 Department of Biomedical Sciences, University of Buea, Cameroon; 9 Department of Microbiology and Immunology, University of Yaoundé I, Yaoundé, Cameroon; International AIDS Vaccine Initiative, UNITED STATES

## Abstract

**Background:**

Little is known about how improved depression care affects HIV-related outcomes in Africa. In a sample of depressed HIV patients in a low income, sub-Saharan country, we explored how implementing measurement-based antidepressant care (MBC) affected HIV outcomes over 4 months of antidepressant treatment.

**Methods:**

As part of a project adapting MBC for use in Cameroon, we enrolled 41 depressed HIV patients on antiretroviral therapy in a pilot study in which a depression care manager (DCM) provided an outpatient HIV clinician with evidence-based decision support for antidepressant treatment. Acute depression management was provided for the first 12 weeks, with DCM contact every 2 weeks and HIV clinician appointments every 4 weeks. We measured HIV clinical and psychiatric outcomes at 4 months.

**Results:**

Participants were moderately depressed at baseline (mean Patient Health Questionnaire [PHQ] score = 14.4, range 13.1, 15.6). All HIV clinical outcomes improved by four month follow-up: mean (range) CD4 count improved from 436 (2, 860) to 452 (132, 876), mean (range) log-viral load decreased from 4.02 (3.86, 4.17) to 3.15 (2.81, 3.49), the proportion with virologic suppression improved from 0% to 18%, mean (range) HIV symptoms decreased from 6.4 (5.5, 7.3) to 3.1 (2.5, 3.7), the proportion reporting good or excellent health improved from 18% to 70%, and the proportion reporting any missed ARV doses in the past month decreased from 73% to 55%. Concurrently, psychiatric measures improved. The mean (range) PHQ score decreased from 14.4 (13.1, 15.6) to 1.6 (0.8, 2.4) and 90% achieved depression remission, while mean maladaptive coping style scores decreased and mean adaptive coping scores and self-efficacy scores improved.

**Conclusion:**

In this pilot study of an evidence-based depression treatment intervention for HIV-infected patients in Cameroon, a number of HIV behavioral and non-behavioral health outcomes improved over 4 months of effective depression treatment. These data are consistent with the hypothesis that better depression care can lead to improved HIV outcomes.

## Introduction

Major Depressive Disorder (MDD) co-occurs frequently with HIV in sub-Saharan Africa, with most studies that use structured interviews reporting a prevalence ranging from 11–38%,[1] a figure roughly two to three times that found in comparable HIV-uninfected populations. [2,3] Depressed HIV-infected patients have higher rates of progression to advanced stages of disease,[2,4] lower CD4 counts, and negative HIV-related behaviors[5] including poor antiretroviral (ARV) adherence,[6,7] unsafe needle sharing,[8,9] and unprotected sex.[9,10] Indeed, global health experts have identified the effects of untreated depression as a matter of public health import. Recent panels have underlined the significance of conducting research on integrating mental health identification and treatment into routine HIV clinical care in less wealthy countries,[11] and the World Health Organization (WHO) identifies mental health treatment as essential care intervention for those living with HIV in resource-limited settings.[12]

However, little health care infrastructure and limited mental health expertise is available to support improved depression care in most of sub-Saharan Africa. The WHO estimates that in low-income countries, three-quarters of those needing mental health treatment lack access to treatment,[13] a gap especially pronounced in sub-Saharan Africa where in 2011 there was a median of 0.05 psychiatrists per 100,000 population, in comparison with a world median of 1.27. [14] Further, no evidence from sub-Saharan Africa addresses whether effective depression management may improve HIV outcomes.

As a resource-efficient, scalable model for medication-based depression care for HIV-infected patients in a resource-limited setting, we adapted and pilot-tested a depression management strategy called measurement-based care (MBC) for application with depressed HIV-infected patients in Cameroon, a low income, sub-Saharan country with minimal mental health services. The model proved feasible, safe, and acceptable; was delivered with high fidelity; and led to substantial reductions in depressive severity over 3 months in nearly all patients.[15] Here we examine the extent to which HIV-related behavioral and health indicators changed among pilot study participants over 4 months of MBC depression treatment.

## Methods

### The Measurement-Based Care Model

Measurement-Based Care (MBC) is an evidence-based, resource-efficient depression management strategy designed to help psychiatric and non-psychiatric medical practitioners to deliver best-practices, guideline-concordant medication management for depression.[16] Consistent with a chronic disease management approach used for other medical illnesses, MBC relies on a non-physician Depression Care Manager (DCM) to regularly and systematically assess key depression treatment measures. The DCM then provides decision support to the treating (non-psychiatric) care provider regarding antidepressant initiation, dosing, duration, and switching. The role of the DCM may be effectively filled by individuals with a range of training, including nurses or social workers, enhancing the adaptability of the model to a variety of clinical settings.

In the MBC model, depressive symptoms and side effect burden are measured at regular intervals with validated instruments and compared to an evidence-based algorithm to produce specific treatment recommendations. The goal is to achieve remission—minimum depressive symptoms with return to pre-depression levels of functioning. MBC offers evidence-based guidance on depression management in situations where access to specialty mental health care is limited. With MBC, guided by a psychiatrist’s clinical supervision, social workers, nurses, or other trained members of the healthcare team can measure symptoms and treatment response, assess tolerability, and provide decision support to non-psychiatric prescribers (e.g., the HIV clinician) based on the treatment algorithm.

We have previously shown in The Sequenced Treatment Alternatives to Relieve Depression (STAR*D) trial, the largest trial of depression treatment, that MBC in primary care settings can produce depression care delivery and outcomes equivalent to that observed in specialty psychiatric clinics.[17] We have also adapted MBC for use in HIV specialty care settings, with particular attention paid to antiretroviral-antidepressant interactions.[16]

### Adaptation

As part of an NIMH-funded grant (“Adaptation of a Depression Treatment Intervention for HIV Patients in Cameroon,” R34 MH084673, 2009–2012), we adapted MBC for use in Cameroon,[18] modifying and validating a culturally-relevant Patient Health Questionnaire 9- item version (PHQ-9)[19] and adapting the algorithm to incorporate the antidepressants available—in this case, amitriptyline.

### Pilot study: Eligibility

Following this adaption, we conducted a pilot study to assess feasibility, acceptability, safety, and preliminary efficacy of the adapted MBC intervention using a single DCM. From April 15-November 30, 2011, HIV-infected patients presenting for care at the Bamenda Day Hospital AIDS Treatment Center were invited to participate in the study if they met the following eligibility criteria: (1) a score ≥ 10 on the PHQ-9[19,20]; (2) current major depressive disorder confirmed through clinical assessment; (3) 18–65 years of age; (4) competent in English. Potential participants were considered not eligible if they met any of the following exclusion criteria: (1) current acute, high-risk suicidality; (2) history of bipolar disorder or psychotic disorder as determined through clinical assessment; (3) current substance abuse problem that in the judgment of the clinician would need to be addressed prior to depression treatment. Potentially eligible patients received information about all study activities, after which interested individuals provided written informed consent.

### Procedures

For all participants enrolling in the pilot study, the DCM provided a recommendation about antidepressant prescription to the treating HIV physician at the time of enrollment. Consistent with the standard MBC timeline,[16,21] the DCM had follow-up contact with each participant every 2 weeks through week 12 post-enrollment (acute phase of treatment), with weeks 4, 8, and 12 being visits with the HIV clinician representing Critical Decision Points (CDPs). At CDP visits, the DCM systematically assessed depressive severity (with the PHQ-9) and antidepressant side effects (with the Frequency, Intensity, and Burden of Side Effects Rating Scale [FIBSER]).[22] Based on these measures and guided by the algorithm, the DCM provided the HIV clinician with a specific treatment recommendation: increase, maintain or decrease the current dose, or switch treatment. Weeks 2, 6, and 10 were interim check-ins to encourage adherence and to monitor and address side effects. Fidelity to this MBC strategy using amitriptyline was high; HIV providers followed MBC recommendations at 96% of encounters.[15] Quality assurance was ensured through weekly supervision of the DCM with a study psychiatrist (BNG), including review of all active cases.

### Outcome measures

Participants completed in-person interviews at enrollment and at 4 months (1 month after the end of acute phase depression treatment) to measure changes in HIV-related behavioral and other health outcomes. Behavioral outcomes included self-reported antiretroviral medication adherence over the past 30 days using a Visual Analog Scale[23] and a single item from the ACTG Adherence Questionnaire[24] asking about the last time the participant missed a dose (within past week; 1–2 weeks ago; 3–4 weeks ago; 1–3 months ago; more than 3 months ago; never miss); problematic alcohol use measured with the AUDIT[25] and the CAGE[26]; and sexual risk behaviors measured with a set of questions asking about frequency of sex with and without condoms in the past month with HIV-infected, HIV-uninfected, and status-unknown partners. For non-behavioral health outcomes, participants self-reported whether in the past 6 months they had experienced each of 13 symptoms commonly associated with HIV infection:[27] new or persistent headaches, fevers, oral pain, white patches in the mouth, rashes, nausea, trouble with eyes, sinus infection, numbness in the hands or feet, persistent cough, diarrhea, weight loss, or (for women only) abnormal vaginal discharge. Participants also self-rated their overall physical health as poor, fair, good, very good, or excellent. Finally, participants provided a blood sample at baseline and 4 months for measurement of CD4 lymphocyte count and HIV RNA viral load (VL).

### Mediators and moderators

We recognized that given the small sample size, we would have limited power to assess mediators and moderators of effect. However, we hypothesized that MBC depression treatment would improve HIV behavioral and health outcomes by reducing depressive severity, which would in turn improve coping styles and self-efficacy (mediators). We measured these mediators with the Patient Health Questionnaire-9 (depressive severity),[19,20] the Brief COPE (adaptive and maladaptive coping styles),[28] and the Watt Self-Efficacy Scale[29] at baseline and 4 months.

Concerning moderators, we hypothesized that we would see larger improvements in HIV-related outcomes among those more severely depressed at baseline compared to those with moderate depression; among early vs. late responders to depression treatment (i.e., comparing those achieving depression remission by 4 vs. 8 vs. 12 weeks); and among those with low vs. high baseline ARV adherence.

### Data analysis

The present analysis of changes in HIV outcomes was restricted to participants already on ART at the time of study enrollment. Sample characteristics and outcome measures are described using proportions for categorical variables and means and range for continuous variables. Differences in means (for continuous variables) and proportions (for categorical variables) were obtained, along with 95% confidence intervals (CIs), comparing the four-month measurements to baseline measurements. Our primary HIV outcomes of interest were change in CD4 lymphocyte count and change in HIV RNA viral load (VL), while our primary psychiatric outcomes of interest were change in PHQ-9 score and proportion with remission of depression.

To assess possible mediation and moderation, we compared the magnitude of change in outcomes between specific subgroups.

Given the small sample size in this pilot study, we focus primarily on magnitude of changes in outcomes rather than tests of statistical significance, and all participants were included in the analysis in an intent-to-treat framework. All analyses were conducted using Stata version 13 (College Station, Texas).

### Ethical approval

All study activities were approved by the Cameroon National Ethics Committee (No. 111/CNE/SE/09), the University of North Carolina at Chapel Hill’s Biomedical Institutional Review Board (IRB) (# 09–0852), and the Duke University Health System IRB (# Pro00016937). The study also received administrative approval from the Ministry of Public Health in Cameroon.

## Results

### Sample Description

**Fig 1:**
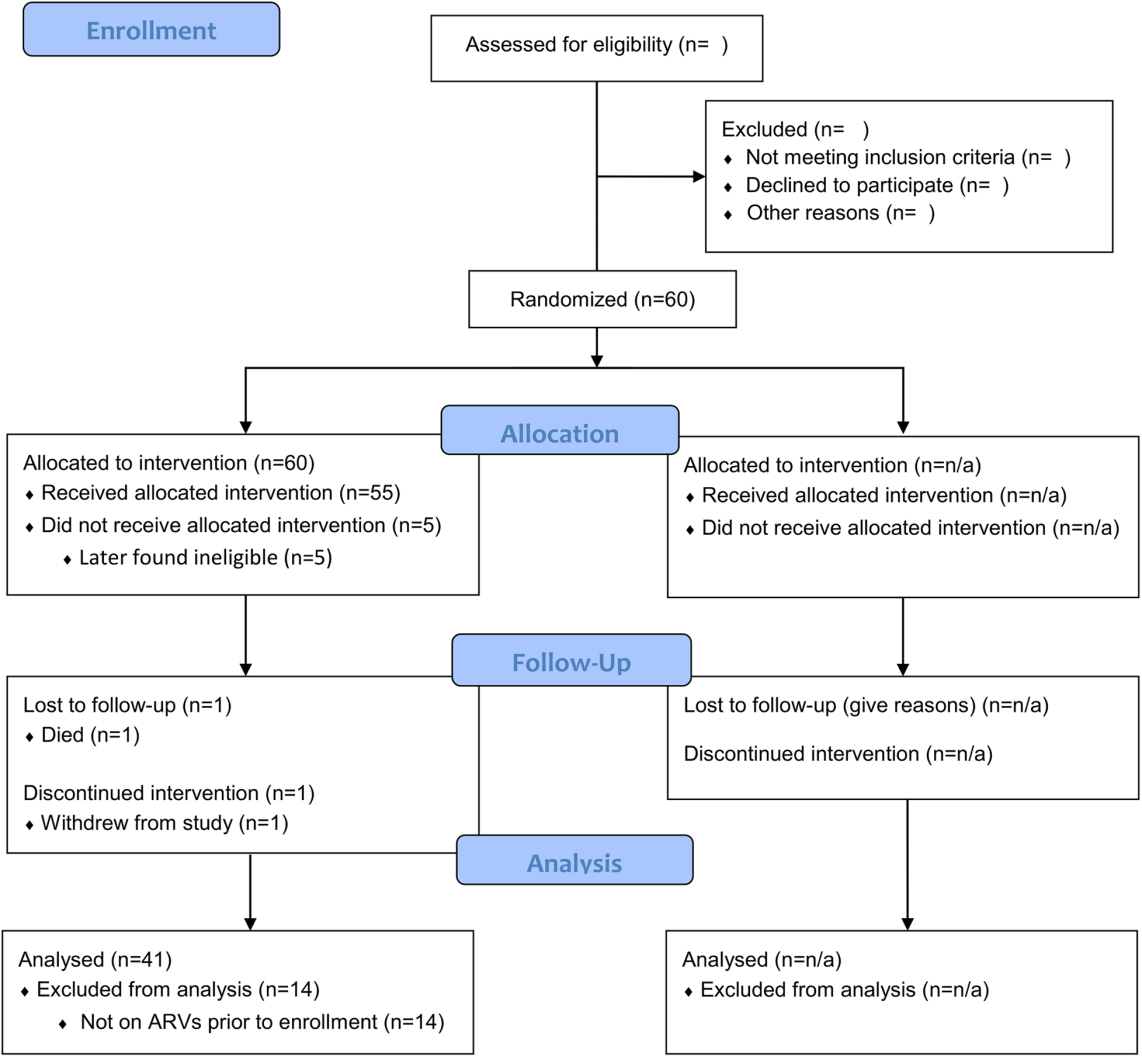
CONSORT 2010 Flow Diagram.

We enrolled 55 depressed HIV participants, of whom 41 were already on ART at the time of enrollment (Fig 1). All further results are restricted to these 41 participants; all results presented below were substantively unchanged if attention was restricted to those on ART ≥3 months (n = 36) or expanded to include all pilot study participants regardless of ART status (n = 55).

Most of those on ART were female (76%) and had attained a primary education only (63%) (Table 1). The majority of participants (88%) were on either nevirapine-based (n = 29, 71%) or efavirenz-based (n = 7, 17%) triple therapy, with a small number (n = 5, 12%) on lopinavir/ritonavir-based triple therapy.

**Table 1:** Description of sample (n = 41).

Characteristic	n (%) or mean (range)
Gender	
Male	10 (24)
Female	31 (76)
Age	39.1 (29, 51)
Marital status	
Married or partnered	5 (12)
Never married	13 (32)
Divorced or widowed	23 (56)
Educational attainment	
Primary	26 (63)
Secondary	11 (27)
Tertiary	4 (10)
Daily expenditures	$5.26 ($0.03, $16.77)
Most common ARV regimens	
Duovir/Nevirapine	16 (29)
Zidovex	11 (20)
Duovir/Stocrine	6 (11)
Baseline PHQ9 score	14.4 (13.1, 15.6)
Moderate (10–14)	24 (59)
Moderately severe (15–19)	11 (27)
Severe (20–27)	6 (15)
Prior depressive episodes	
None	1 (2)
1	4 (10)
2	14 (34)
3 or more	21 (54)
Ever treated for depression	1 (2)

The mean (range) baseline PHQ-9 was 14.4 (13.1, 15.6), consistent with moderate depressive severity (PHQ-9 10–14). Depression was likely to be recurrent; 34% had two prior episodes, and 54% had three or more prior episodes. Only 2% (1 participant) had ever been treated for depression (Table 1).

Participants reported a mean (range) of 6.4 (5.5, 7.3) HIV-related symptoms in the past week, and 18% described their overall physical health as good, very good, or excellent, at baseline. Clinical HIV measures showed a mean (range) baseline CD4 count of 436 (2, 860) cells/mm^3^, and mean (range) log_10_ VL of 4.02 (3.86, 4.17) copies/mL, (Table 2). Of note, no participants met criteria for viral suppression at baseline (VL < 400 copies/mL).

**Table 2:** Clinical Outcomes.

	Baseline n (%) or Mean (range)	4 months n (%) or Mean (range)	Proportion or Mean Difference (95% CI)
Active participants	41	40	
Completed interview	41 (100%)	40 (100%)	
*Clinical outcomes*			
CD4 count (cells/mm3)	436 (2, 860)	452 (32, 876)	16 (-47, 79)
Log-10 Viral load	4.02 (3.86, 4.17)	3.15 (2.81, 3.49)	-0.87 (-1.21, -0.53)
% <400 copies/mL	0 (0)	5 (18%)	18% (4%, 32%)
HIV-related symptoms	6.4 (5.5, 7.3)	3.1 (2.5, 3.7)	-3.3 (-4.1, -2.5)
Self-reported health good to excellent	7 (18%)	28 (70%)	0.53 (0.34, 0.71)
% self-rating improved		32 (80%)	
% self-rating remained the same		4 (10%)	
% self-rating declined		2 (5%)	

Self-reported ARV adherence at baseline was relatively high, with over half reporting ARV adherence of 95% or greater in the past month (Table 3). However, 73% of participants at baseline reported missing at least one dose in the past month on either the VAS or the ACTG single-item adherence question.

**Table 3:** Behavioral Outcomes, Past Month.

	Baseline n (%)	4 months n (%)	Proportion Difference (95% CI)
*Self-reported ARV adherence*			
Adherence 95% or greater	21 (53%)	25 (63%)	10% (-11%, 32%)
Missed any doses	29 (73%)	22 (55%)	-18% (-38%, 3%)
*Alcohol use*			
AUDIT categorization			
No use	21 (53%)	31 (78%)	25% (5%, 45%)
Low-risk use (1–7)	16 (40%)	9 (23%)	-18% ('37%, 2%)
Hazardous use (8 or more)	3 (8%)	0	-8% (-16%, 1%)
CAGE—score of 2 or more (clinically sig.)	7 (18%)	4 (10%)	-8% (-23%, 8%)

Participants had a median of one sexual partner in the past month, and 7% reported having any unprotected sex in the past month. Alcohol use overall was low (Table 3).

During the course of the project, 1 of the 41 participants, with a history of HIV-associated nephropathy, died of acute renal failure and pyelonephritis unrelated to study participation shortly after completing her week 4 visit. For the remaining 40 participants, there were no losses to follow-up at 4 months.

### HIV Clinical Outcomes at 4-month Follow-up

All HIV clinical outcomes, including our two primary outcomes, had improved by 4-month follow-up, although by varying degrees (Table 2). CD4 count improved, with a mean difference (95% CIs) of 16 (-47, 79), while (log) VL improved more noticeably, with a mean difference (95% CI) of -0.87 (-1.21, -0.53). Notably, 18% of participants had newly achieved virologic suppression by 4 months. Other outcomes also suggested benefit. The mean number of self-reported HIV symptoms decreased by 3.3 (95% CI 2.5, 4.1). The proportion reporting good, very good, or excellent health improved nearly four-fold, with a mean percentage change (95% CI) of 53% (34%, 71%), and 80% reported an overall improvement in health.

Measurement of behavioral outcomes suggested some improvement in adherence (Table 3). The proportion of participants reporting 95% or greater adherence on the VAS increased by 10% (95% CI -11%, 32%) to 63%, and the proportion reporting any missed ARV doses on either the VAS or the ACTG single-item question decreased from 73% to 54%. Mean self-reported adherence on the VAS did not substantially change.

### Psychiatric Measures at 4-month Follow-up

All psychiatric measures were consistent with improved outcomes (Table 4). Our two primary psychiatric outcomes showed benefit. The PHQ-9 score decreased significantly (mean difference (95% CI) -12.8 (-14.2, -11.3)), and 90% of participants achieved remission (PHQ-9 < 5). Secondary outcomes also suggested improvement. The mean adaptive coping style increased from 3.0 to 3.2 (mean difference (95% CI) 0.2 (0.0, 0.3)), the mean maladaptive coping style score decreased from 1.7 to 1.5 (mean difference (95% CI) 0.2 (0.1, 0.4)), and self-efficacy increased from 3.5 to 3.7 (mean difference (95% CI) 0.2 (0.0, 0.3) (all on a 1–4 scale).

**Table 4:** Changes in Hypothesized Mediators.

	Baseline n (%) or Mean (SD)	4 months n (%) or Mean (SD)	Proportion or Mean Difference (95% CI)
Active participants	41	40	
Completed interview	41 (100%)	40 (100%)	
*Depressive outcomes*			
PHQ-9 total score	14.4 (13.1, 15.6)	1.6 (0.8, 2.4)	-12.8 (-14.2, -11.3)
Remission (PHQ-9 < 5)	0 (0%)	36 (90%)	90% (81%, 99%)
Adaptive coping styles (mean score)	3.0 (2.8, 3.2)	3.2 (3.0, 3.3)	0.2 (0.0, 0.3)
Maladaptive coping styles (mean score)	1.7 (1.5, 1.9)	1.5 (1.3, 1.6)	0.2 (0.1, 0.4)
Self-efficacy	3.5 (3.3, 3.7)	3.7 (3.6, 3.8)	0.2 (0.0, 0.3)

### Potential Moderators/Mediators of Response

In exploratory analyses, we assessed whether particular variables might be moderators or mediators of outcomes. These analyses were necessarily preliminary given the small sample size.

When considering moderation or mediation of changes in ARV adherence, there was greater improvement in the proportion reporting >95% adherence among those with severe depression at baseline (50 percentage point improvement) than among those with moderate to moderately severe (3 percentage point improvement), and greater improvement among those with low baseline adherence (36 percentage point improvement) than among those with high baseline adherence (no change). There was also evidence of moderation in the anticipated direction by time to depression treatment response.

When considering moderation or mediation of achievement of virologic suppression, there was little evidence of difference by baseline depressive severity or coping style. There was some suggestion that faster remission of depression was associated with greater likelihood of achieving suppression.

## Discussion

In this pilot study of an evidence-based depression treatment intervention for HIV-infected patients in Cameroon, nearly all participants experienced a strong improvement in their depressive illness. Meanwhile, over the course of 4 months of depression treatment, a number of HIV behavioral and health outcomes also improved. Among study participants, mean viral load decreased by one log and the proportion with virologic suppression improved from 0% to 19% over 4 months. Self-reported HIV symptoms, overall health, and ARV adherence all improved by varying amounts. Hence, HIV improvement paralleled depression improvement, and was consistent with a model in which effective depression care yields improved HIV outcomes.

This is the first study to our knowledge to provide evidence, even if only suggestive, that depression treatment may lead to improved HIV clinical outcomes among depressed HIV-infected individuals in sub-Saharan Africa. An increasing number of studies identify the high prevalence of depression among those living with HIV in Africa as well as the consistent associations between depression and low antiretroviral adherence. These findings mirror the much deeper literature on the prevalence and negative consequences of depression among people living with HIV in high-income countries. Some,[30] although not all,[31] studies in the US have suggested that treating depression may improve HIV outcomes, but no such studies to our knowledge have been reported yet from sub-Saharan Africa where both the prevalence of HIV and the barriers to providing mental health care are much greater. Also, these findings are consistent with the sparse literature in non-clinical trial observational cohort studies showing an association between antidepressant treatment and improvements in adherence[30,32,33] and viral control,[32] and with recent retrospective chart data showing a correlation between depression remission and undetectable viral loads.[34]

Although the preliminary nature of this study precludes any firm conclusions about the relationship between depression treatment and HIV outcomes, exploratory analyses provide some clues that treating the most severely depressed may offer marked benefits. For example, the greatest gains in ARV adherence were achieved by the most severely depressed group, who started out with the worst adherence but by 4 months had the best adherence (relative to those with moderately severe or moderate depression at baseline). Time to depression remission might also mediate virologic suppression, with a dose-response relationship observable between the time to depression remission and the likelihood of achieving virologic suppression by 4 months.

There are limitations to our findings. First, this pilot study did not include a comparison group, so we cannot account for depression that would have recovered without active depression treatment. However, nearly all participants reported chronic or recurrent depression that would be unlikely to remit so uniformly on its own in the absence of treatment, and a treatment effect is further supported by simultaneous improvement in several other psychiatric measures including suicidal ideation, self-efficacy, and coping styles. Second, the sample size is small, precluding any firm conclusions about the roles of moderators and mediators of the relationship between depression improvement, ARV adherence, and HIV outcome. Third, while associated, the improvement in both depression and HIV outcomes may not be causally related. These findings are clearly preliminary. For example, HIV clinical markers may have improved due to antiretroviral treatment alone; however, nearly all participants were on stable antiretroviral therapy at the time of enrollment rather than newly initiating therapy.

In summary, these data support a conceptual model in which successful depression treatment in 4–12 weeks might lead to a decreased maladaptive coping style and improved self-efficacy, resulting in improvement in ARV adherence and increased likelihood of virologic suppression. The intervention may be especially beneficial in those with more severe depression and those who achieve early remission of their depression. This pilot study provides some of the first evidence, even if only suggestive, in sub-Saharan Africa that effective depression treatment may play an important role in optimizing HIV treatment benefits among HIV-infected patients with depression. In this sample of depressed HIV-infected patients in Cameroon receiving evidence-based depression care management, both depressive and HIV outcomes improved over 4 months of evidence-based depression treatment. These data are consistent with the hypothesis that better depression care can lead to improved HIV outcomes. Subsequent large scale prospective trials can test whether these relationships hold true.
